# Biobanks: a basis for quality research

**DOI:** 10.31744/einstein_journal/2020CE5266

**Published:** 2019-12-10

**Authors:** Igor Carreiro Ramalho, Carlos Magno da Costa Maranduba

**Affiliations:** 1 Universidade Federal de Juiz de Fora, Juiz de Fora, MG, Brazil.

Dear Editor,

Considering that today there is a growing concern with the quality of cells, tissues, and organs destined for the treatment of human diseases, within this context, many studies have been conducted by public institutions. Several organizations store biological samples and data, given the difficulty to obtain and characterize many of these materials, as they can possibly be used in new projects. Based on the current legislation, storage, as mentioned, characterizes a biobank.^(^^1^^)^ These structures require investment for maintenance of the samples stored. Today we have private units that have their own resources for deposits of private samples, with a limited use of the samples, in addition to public biobanks, in which the certified samples can be used for different purposes, guaranteeing the benefit of their serviceability; for those destined to research, carrying out projects with samples and/or quality biological data.^(^^2^^–^^4^^)^

Today, Brazil has 53 regulated biobanks and a few others in course.^(^^1^^)^ Observing the world scenario as to human biological samples, the United States has 169 biobanks, while Europe has 90, and Asia, 23. There are yet 26 additional biobanks for storage of samples of animals and plants distributed over 12 countries.^(^^5^^)^ In face of this world scenario, it is crucial that competent authorities understand the need for and the importance of investments in this sector, in order to improve the excellence of research done in Brazil. The lack of investments in these structures directly impacts the quality of the outcome of products and services that society looks forward to.^(^^6^^)^ Therefore, it is vital that Brazil have its position set in this scenario, with biobanks of high structural and operational standards, placing our country on equal terms with developed countries.
