# Supplementary material for: Biobanks: a basis for quality research
Source: Einstein (Sao Paulo). 2019 Dec 10;18:eCE5266. doi: 10.31744/einstein_journal/2020CE5266 (PMC6910064; doi:10.31744/einstein_journal/2020CE5266)
Supplement: Translation

# Translation: Biobancos: uma base para pesquisas de qualidade

**Authors:** Igor Carreiro Ramalho, Carlos Magno da Costa Maranduba

**Affiliations:** 1 Universidade Federal de Juiz de Fora, Juiz de Fora, MG, Brasil.

Caro Editor,

Visto que existe hoje uma preocupação crescente com a qualidade das células, tecidos e órgãos voltados para tratamento de enfermidades humanas, muitas pesquisas nesse contexto vêm sendo realizadas por instituições públicas. Várias instituições armazenam amostras e dados biológicos, dada a dificuldade de obtenção e caracterização de muitos desses materiais, sendo que os mesmos apresentam possibilidade de utilização em novos projetos. Com base na legislação vigente, o armazenamento, como mencionado, caracteriza um biobanco.^(^^1^^)^ Essas estruturas requerem investimentos para manutenção das amostras arquivadas; hoje dispomos de unidades privadas, que possuem recursos próprios para depósitos de amostras particulares, tendo uso limitado das mesmas, além de biobancos públicos, nos quais as amostras certificadas podem ser utilizadas com diferentes fins, garantindo o benefício de sua operacionalidade; para aqueles com destino às pesquisas, a realização de projetos com amostras e/ou dados biológicos de qualidade.^(^^2^^–^^4^^)^

O Brasil hoje conta com 53 biobancos regularizados e alguns outros em tramitação.^(^^1^^)^ Observando o cenário mundial, no que diz respeito às amostras biológicas humanas, os Estados Unidos possuem 169 biobancos, enquanto a Europa tem 90 e a Ásia outros 23. Ainda existem outros 26 biobancos para armazenamento de amostras de animais e plantas distribuídos em 12 países.^(^^5^^)^ Diante desse cenário mundial, é indispensável que as autoridades competentes entendam a necessidade e a importância de investimentos nesse setor, para melhorar a excelência das pesquisas realizadas no Brasil. A falta de investimentos nessas estruturas impacta diretamente na qualidade do retorno de produtos e serviços pelos quais a sociedade anseia.^(^^6^^)^ Portanto, é vital que o Brasil tenha sua posição firmada nesse cenário, possuindo biobancos com altos padrões estruturais e operacionais, colocando nosso país em pé de igualdade com os países desenvolvidos.
